# Trajectory of mid-arm subcutaneous fat, muscle mass predicts mortality in hemodialysis patients independent of body mass index

**DOI:** 10.1038/s41598-024-64728-8

**Published:** 2024-06-18

**Authors:** Yuqi Yang, Qian Li, Wanting Qiu, Helin Zhang, Yuyang Qiu, Jing Yuan, Yan Zha

**Affiliations:** 1 School of Basic Medicine, Guangzhou Medical University, Guangzhou, China; 2 Department of Nephrology, Guizhou Provincial People’s Hospital, Zhongshan East Road, Guiyang, China

**Keywords:** Triceps skinfold thickness, Mid-upper arm circumference, Trajectory, Mortality, Hemodialysis, Cardiology, Medical research, Nephrology, Risk factors

## Abstract

Although decreasing body mass index (BMI) is associated with higher mortality risk in patients undergoing hemodialysis (HD), BMI neither differentiates muscle and fat mass nor provides information about the variations of fat distribution. It remains unclear whether changes over time in fat and muscle mass are associated with mortality. We examined the prognostic significance of trajectory in the triceps skinfold (TSF) thickness and mid-upper arm circumference (MUAC). In this multicenter prospective cohort study, 972 outpatients (mean age, 54.5 years; 55.3% men) undergoing maintenance HD at 22 treatment centers were included. We calculated the relative change in TSF and MUAC over a 1-year period. The outcome was all-cause mortality. Kaplan–Meier, Cox proportional hazard analyses, restricted cubic splines, and Fine and Gray sub-distribution hazards models were performed to examine whether TSF and MUAC trajectories were associated with all-cause mortality. During follow-up (median, 48.0 months), 206 (21.2%) HD patients died. Compared with the lowest trajectory group, the highest trajectories of TSF and MUAC were independently associated with lower risk for all-cause mortality (HR = 0.405, 95% CI 0.257–0.640; HR = 0.537; 95% CI 0.345–0.837; respectively), even adjusting for BMI trajectory. Increasing TSF and MUAC over time, measured as continuous variables and expressed per 1-standard deviation decrease, were associated with a 55.7% (HR = 0.443, 95% CI 0.302–0.649), and 97.8% (HR = 0.022, 95% CI 0.005–0.102) decreased risk of all-cause mortality. Reduction of TSF and MUAC are independently associated with lower all-cause mortality, independent of change in BMI. Our study revealed that the trajectory of TSF thickness and MUAC provides additional prognostic information to the BMI trajectory in HD patients.

## Introduction

End-stage kidney disease (ESKD) affects approximately 2 million people globally ^1^, and approximately 69% of patients with ESKD are treated with hemodialysis (HD), which is one of the most widely used renal replacement therapies ^2^. Despite advances in dialysis technology, these patients face a high risk of mortality, and the annual report on kidney disease from China shows that the mortality rate of HD patients reaches 12.5%, imposing a substantial burden on patients and the healthcare system ^3^. Cardiovascular disease (CVD) is the most common cause of death, accounting for approximately 50% ^3,4^.

The “Obesity paradox” phenomenon has been investigated in HD patients from several large cohort studies in the past decades ^5^. Obesity is a traditional risk factor for mortality in the general population ^6,7^. By contrast, obesity is inversely associated with better survival among HD patients ^8^. Although obesity is generally defined by body mass index (BMI), some studies have stated that BMI is an imperfect predictor of mortality ^9,10^, considering that BMI could not give a reliable assessment of body composition, especially could not differentiate muscle mass from fat mass ^11^. Anthropometric measurements are simple, noninvasive, and cost-effective techniques, and have been widely used to evaluate body composition. As indicators of mid-arm measurements, triceps skinfold (TSF) thickness determines subcutaneous adipose tissue ^12^, and mid-upper arm circumference (MUAC) reflects the amount of muscle mass of the mid-arm ^13^, which is superior to BMI in predicting nutritional status. Previous studies have found that they were inversely associated with all-cause mortality, independent of BMI, in the general population ^14,15^, including HD patients ^16,17^. However, changes in body composition constantly occur in HD patients due to a variety of factors, including decreased protein and energy intake, hormonal changes, and deterioration of the water and sodium metabolism ^18^. Therefore, prognostic values of anthropometric measurements for mortality may also change over time. Most prior studies have only evaluated the effect of baseline mid-arm measurements, obtained at the start of the study, on subsequent mortality. Recently, several studies have reported that changes in mid-arm measurements are significantly related to associated with increased risk of mortality in general populations ^19–21^. However, whether the mid-arm change over time can be used as a prognostic tool remains unclear in HD patients.

From these perspectives, we hypothesized that a decrease in the mid-arm measurements over time would be associated with a high risk of mortality among HD patients. Therefore, the present study assessed mid-arm measurement trajectory over 1 year to investigate their associations with all-cause mortality in patients undergoing maintenance HD.

## Methods

### Ethics statement

All protocols were approved by the ethics committee of Guizhou Provincial People’s Hospital (approval number: (2020)208) and adhered to the principles of the Declaration of Helsinki. All participants provided written informed consent before participation. Trained research doctors performed the baseline and follow-up assessments through face-to-face questionnaire interviews and physical measurements.

### Study design and participants

This multicenter prospective cohort study was conducted in 22 HD units in Guizhou Province, China. This study recruited patients who underwent maintenance HD therapy in these HD units from June 1, 2015, to September 30, 2016. Patients were excluded if they were younger than 18 years, had HD treatment for less than three months, missed mid-arm measurement data at baseline and/or one year later, withdrew from HD, death, and lost to follow-up before the end of the one year. All the patients performed HD with conventional dialyzers under the standard temperature (35.5–36.5 °C). The dialysate composition is usually composed of sodium (130–140 mmol/L), potassium (3–4 mmol/L), chloride (96–110 mmol/L), calcium (1.5–1.75 mmol/L), magnesium (0.6–1.0 mmol/L), bicarbonate (32–38 mmol/L). The electrolyte concentrations would be adjusted accordingly. The dialysis modes were decided according each HD unit.

### Anthropometric measurements

Mid-arm measurements were performed on the non-fistula arm by trained workers following standardized protocols for anthropometric measurements, as recommended by the World Health Organization. TSF thickness was measured at the mid-point of the posterior line between the olecranon and the tip of the acromion using skinfold calipers and recorded to the nearest 0.5 mm. MUAC was measured at the mid-point of the mid-upper arm with the elbow fully extended, and results were recorded to the nearest 0.1 cm. The mid-point of the mid-upper arm was defined as the midway between the olecranon process of the ulna and the acromion process of the scapula, which was located after bending the right arm to a 90° angle at the elbow.

Mid-arm muscle circumference (MAMC) was derived from the equation ^22^:

$${\text{MAMC}}\left( {{\text{centimeters}}} \right)\, = \,{\text{MUAC }}\left( {{\text{centimeters}}} \right) - \pi \, \times \,({\text{TSF}}\;{\text{ thickness}}\left[ {{\text{centimeters}}} \right]).$$

Other anthropometric measurements. Weight was measured using a calibrated beam scale with the participant wearing lightweight clothing, and height was measured without shoes using a portable stadiometer. BMI was calculated as weight (kilograms) divided by height (meters) squared (overweight ≥ 24 kg/ m^2^). Waist circumference (WC) was measured at a point midway between the lowest rib and the iliac crest in a horizontal plane, and hip circumference was measured using non-plastic tape (central obesity ≥ 90 cm for males, and ≥ 85 cm for females). Waist-height ratio (WHtR) was calculated as WC (centimeters) divided by height (centimeters).

We calculated TSF trajectory from the difference in TSF over the first 1 year after randomization as (TSF at 1 year visit—baseline TSF)/baseline TSF, expressed as a percentage. MUAC, BMI, and WHtR trajectories were measured similarly. The values for trajectories in mid-arm measurements (TSF and MUAC) were divided into four categories according to quartiles of changes in TSF and MUAC, respectively.

The cutoff of TSF and MUAC trajectories were determined by the median values, respectively. The selected thresholds were then used to define the C1 (≤ cutoff) and C2 (> cutoff) groups. The survival analysis was further performed in the groups cross-classified with trajectories of TSF and MUAC. According the cutoff group, patients were categorized into four group: T1 (C1 of both TSF and MUAC), T2 (C2 of TSF and C1 of MUAC), T3 (C1 of TSF and C2 of MUAC), and T4 (C2 of both TSF and MUAC).

### Clinical covariate measurements

Information on demographic characteristics, obtained via a questionnaire, included age, sex, and educational level (high: ≥ 12th or low: < 12th). Lifestyle factors were collected via the questionnaire and included smoking status (current/ever smoker or never), alcohol consumption (yes or no), and living status (living alone or with family). Blood pressure was measured after rest for 10 min in the seated position using standard sphygmomanometers before the HD treatment. Comorbidities were scored the modified Charlson comorbidity index (mCCI) for each HD patient, based on ICD-10 diagnostic codes ^23^. All patients were evaluated with the malnutrition-inflammation score (MIS), a specifical nutritional scoring system of evaluating malnutrition and inflammation for dialysis patients, through questionnaire ^24^. Laboratory data within 1 month before the anthropometric assessment were collected from the medical records, including hemoglobin, albumin, serum creatinine, calcium, phosphorus, uric acid, triglyceride, total cholesterol, parathyroid hormone (PTH), high density lipoprotein-cholesterol (HDL-c), and low density lipoprotein-cholesterol (LDL-c).

### Follow-up and outcomes

The primary endpoint was all-cause mortality. Participant death, the exact time of death, and the cause of death were identified from reports from each HD unit in each survey. The baseline for each participant was set as the survey day of the first entry into the survey with complete mid-arm measurements. The observation period was basically until either death, transfer to kidney transplantation, peritoneal dialysis, loss to follow-up, or the end of the study on September 30, 2022.

### Statistical analysis

Participant baseline characteristics were described as a number (percentage) for categorical variables and means (SD) or as a median (interquartile range) for continuous variables. Normally distributed variables were expressed using mean ± standard deviation, whereas non-normally distributed variables were expressed as median and interquartile range. The differences among quartile groups divided by TSF, and MUAC trajectories were compared using one-way analysis of variance or the Kruskal–Wallis test for continuous variables and the chi-squared test for categorical variables.

The associations between baseline and trajectory of BMI, TSF, and MUAC were analyzed with linear regression. Kaplan–Meier survival curves were used to assess the associations between trajectories of mid-arm measurements and mortality, with the log-rank test examining the significant differences between different groups. Cox proportional hazards models were used to calculate hazard ratios (HRs) and 95% confidence intervals (CIs) for all-cause mortality, and multiple covariates were included in multivariable-adjusted models. TSF and MUAC trajectories were analyzed as continuous variables (per SD increment), quartile variables, and threshold values. Model 1 was adjusted for age, sex, educational level, living status, smoking status, and alcohol consumption. Model 2 was adjusted for the variables in Model 1 as well as systolic blood pressure, diastolic blood pressure, and diabetes, hypertension, cardiovascular disease, dialysis frequency, hemoglobin level, albumin level, creatinine level, MIS, mCCI, and BMI trajectory. Model 3 was adjusted for the variables in Model 2 as well as baseline BMI, TSF, and MUAC. To examine the independent association between TSF and MUAC trajectories, a mutually adjusted model was created by including both MUAC and TSF change as well as the covariates in Model 3. In order to model the shape of associations with flexibility and to test for linearity, restricted cubic splines (RCS) with three knots at the 10th, 50th, and 90th centiles were used for TSF and MUAC trajectories in multivariable-adjusted models.

Possible modifiers of the association between TSF, MUAC trajectories with the risk of all-cause mortality were assessed for variables including age (< 65 years or ≥ 65 years), sex (male or female), diabetes (yes or no), BMI (Overweight or no), WC (central obesity or no) at baseline, and MUAC/TSF trajectory, respectively. The heterogeneity between groups was determined using the *P* value for heterogeneity, which was calculated with multiplicative terms by multiplying mid-arm measurements by continuous variables used in the multivariable model.

Additionally, as kidney transplantation was a competing risk event against death, the cumulative incidence considering the competing risk was compared using Gray’s test, and the Fine and Gray sub-distribution hazards model was used in the multivariate model as a sensitivity analysis method for the outcome, together with the standard Cox regression model for cause-specific hazards.

All statistical analyses were performed with R version 4.2.2 (www.r-project.org/), and *P* < 0.05 (two-sided) was considered statistically significant.

### Ethical standards

Ethical approvals were obtained from Guizhou Provincial People’s Hospital-Research Ethics Committees (Approval number: (2020)208). All participants provided written informed consent and all research procedures were conducted in accordance with relevant guidelines and regulations.

## Results

### Characteristics of participants

**Figure 1:**
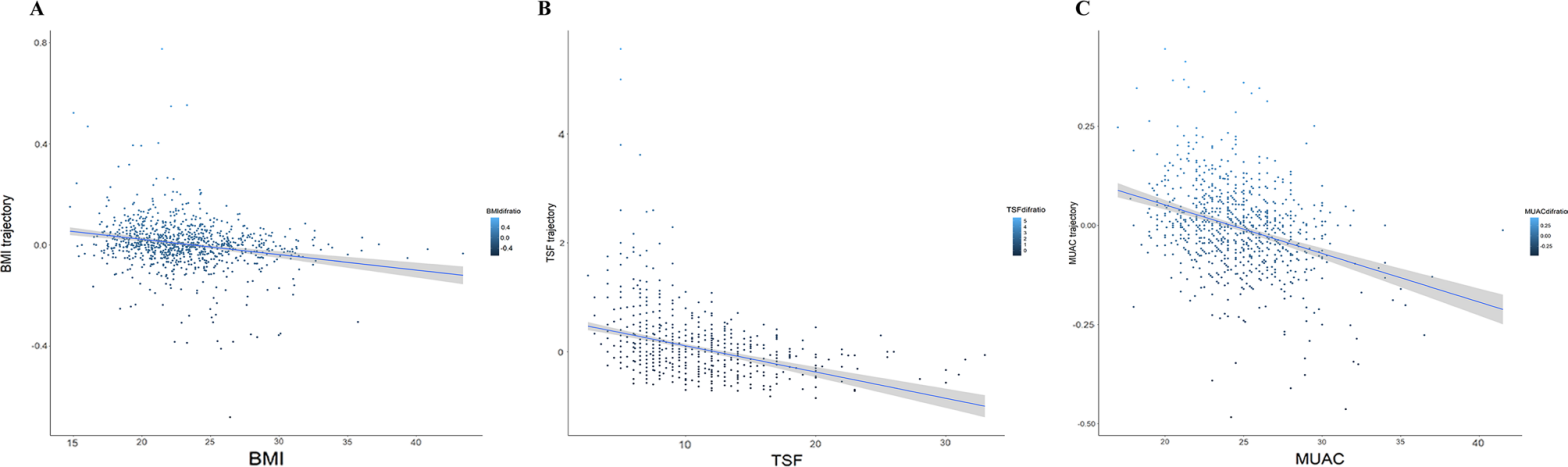
Scatter diagram for the associations between baseline and trajectory of BMI (**A**), TSF (**B**), and MUAC (**C**). BMI, body mass index; TSF, triceps skinfold; MUAC, mid-upper arm circumference.

A total of 1119 HD patients were identified at baseline. Within the first follow-up year, 13 patients died, 36 patients withdraw HD therapy, 52 patients lost to follow-up, and 46 patients had no TSF or MUAC measurement data. These patients were excluded, and finally, 972 patients (mean [SD] age: 54.5 [14.9] years; 538 [55.3%] male) were analyzed finally (Figure S1). 325 (33.4%) patients had a history of diabetes, 816 (84.0%) had hypertension and 213 (21.9%) had CVD. Overall, baseline TSF thickness was 10.0 ± 4.2 mm, and 1-year percent change in TSF was 0.1 (− 25.5 to 30.4) %; baseline MUAC was 24.6 ± 2.9 cm, and 1-year percent change in TSF was − 0.4(− 6.1, 5.0) %. Additionally, patient characteristics at baseline are shown in Table 1. The associations between baseline and trajectories of BMI, TSF, and MUAC are shown in Fig. 1A-C, respectively.

**Table 1:** characteristics of the entire cohort and stratified according to quartiles of TSF and MUAC trajectories among HD patients.

Characteristics	Overall	TSF trajectory quartiles, %				*P*	MUAC trajectory quartiles, %				*P*
		Q1 (≤ − 25.6)	Q2 (− 25.6 to 0.1)	Q3 (0.1 to 30.6)	Q4 (≥ 30.6)		Q1 (≤ − 6.1)	Q2 (− 6.1 to − 0.4)	Q3 (− 0.4 to 5.1)	Q4 (≥ 5.1)	
Number	972	243	243	245	241		243	243	247	239	
Demographic											
Age, year	54.5 ± 14.9	53.6 ± 15.2	55.2 ± 14.7	53.9 ± 14.5	55.4 ± 15.0	0.436	56.4 ± 14.8	53.6 ± 14.5	54.0 ± 14.7	54.0 ± 15.4	0.165
Male, n (%)	537 (55.2%)	145 (59.7%)	140 (57.6%)	129 (52.7%)	123 (51.0%)	0.182	137 (56.4%)	137 (56.4%)	133 (53.8%)	130 (54.4%)	0.915
Low educational level, n (%)	676 (69.5%)	169 (69.5%)	177 (72.8%)	170 (69.4%)	160 (66.4%)	0.497	168 (69.1%)	169 (69.5%)	179 (72.5%)	160 (66.9%)	0.620
Ever/current smoker, n (%)	321 (33.0%)	88 (36.2%)	78 (32.1%)	77 (31.4%)	78 (32.4%)	0.673	81 (33.3%)	75 (30.9%)	82 (33.2%)	83 (34.7%)	0.840
Alcohol consumers, n (%)	36 (3.7%)	12 (4.9%)	6 (2.5%)	9 (3.7%)	9 (3.7%)	0.556	6 (2.5%)	1 (0.4%)	9 (3.6%)	20 (8.4%)	< 0.001
Living alone, n (%)	686 (70.6%)	165 (67.9%)	168 (69.1%)	186 (75.9%)	167 (69.3%)	0.200	150 (61.7%)	175 (72.0%)	179 (72.5%)	182 (76.2%)	0.004
SBP (mmHg)	137.6 ± 20.3	138.4 ± 21.8	137.5 ± 19.4	137.2 ± 19.9	137.6 ± 20.2	0.920	137.9 ± 20.0	136.5 ± 20.8	138.9 ± 20.2	137.4 ± 20.3	0.622
DBP (mmHg)	76.6 ± 25.4	75.7 ± 13.9	75.5 ± 11.7	77.9 ± 12.5	77.2 ± 15.9	0.672	74.7 ± 12.8	76.0 ± 12.3	79.2 ± 45.1	76.5 ± 13.8	0.257
Medical history											
Diabetes, n (%)	325 (33.4%)	82 (33.7%)	75 (30.9%)	74 (30.2%)	94 (39.0%)	0.155	81 (33.3%)	76 (31.3%)	90 (36.4%)	78 (32.6%)	0.664
Hypertension, n (%)	816 (84.0%)	204 (84.0%)	205 (84.4%)	198 (80.8%)	209 (86.7%)	0.363	196 (80.7%)	211 (86.8%)	212 (85.8%)	197 (82.4%)	0.211
CVD, n (%)	213 (21.9%)	59 (24.3%)	42 (17.3%)	54 (22.0%)	58 (24.1%)	0.213	50 (20.6%)	58 (23.9%)	54 (21.9%)	51 (21.3%)	0.839
mCCI	6.6 ± 2.1	6.6 ± 2.2	6.7 ± 2.0	6.6 ± 2.0	6.6 ± 2.0	0.962	6.6 ± 2.1	6.6 ± 2.0	6.5 ± 2.1	6.5 ± 2.1	0.938
Dialysis-related											
HD thrice/week, n (%)	743 (76.4%)	175 (72.0%)	191 (78.6%)	189 (77.1%)	185 (76.8%)	0.352	182 (74.9%)	193 (79.4%)	181 (73.3%)	184 (77.0%)	0.415
HD duration	84.0 [61.0, 110.0]	85.0 [59.0, 117.5]	84.0 [61.3, 105.8]	82.5 [61.0, 106.0]	85.5 [61.3, 109.8]	0.676	84.5 [63.0, 114.0]	89.0 [62.0, 116.0]	84.0 [60.0, 108.0]	75.0 [60.0, 102.0]	0.007
Laboratory											
Hemoglobin, mmHg	105.9 ± 20.5	107.4 ± 20.5	104.5 ± 18.7	106.1 ± 21.4	106.1 ± 21.6	0.490	105. 7 ± 19.9	106.4 ± 19.9	107.1 ± 21.7	104.6 ± 20.3	0.567
Albumin, g/L	40.1 ± 5.6	40.7 ± 4.9	39.8 ± 5.5	40.0 ± 5.9	39.9 ± 6.2	0.340	40.0 ± 5.24	39.5 ± 6.2	40.6 ± 5.0	40.3 ± 5.9	0.214
Creatinine, umol/L	850.4 ± 335.6	872.4 ± 359.6	853.4 ± 323.1	875.87 (341.18)	802.0 ± 316.3	0.073	827.3 ± 311.5	868. 8 ± 337.0	886.8 ± 351.8	817.7 ± 336.9	0.079
Calcium, mmol/L	2.2 ± 0.3	2.2 ± 0.3	2.1 ± 0.3	2.2 ± 0.3	2.2 ± 0.3	0.350	2.2 ± 0.3	2.2 ± 0.3	2.2 ± 0.3	2.1 ± 0.3	0.041
Phosphorus, mmol/L	1.7 ± 0.7	1.8 ± 0.9	1.7 ± 0.6	1.7 ± 0.7	1.6 ± 0.7	0.023	1.7 ± 0.9	1.7 ± 0.7	1.8 ± 0.7	1.7 ± 0.7	0.439
PTH, pg/mL	332.2 [176.8, 645.6]	350.3 [200.8, 718.7]	342.7 [183.1, 623.5]	318.5 [149.4, 633.9]	311.2 [173.2, 633.3]	0.610	370.4 [212.3, 760.1]	343.8 [176.0, 662.0]	305.3 [152.5, 662.0]	310.3 [176.8, 580.7]	0.194
Uric acid, umol/L	414.7 ± 130.5	413.0 ± 137.6	430.1 ± 123.8	425.5 ± 134.2	388.8 ± 124.2	0.003	408.0 ± 120.8	416.9 ± 135.4	414.2 ± 122.6	419.6 ± 141.8	0.801
Triglyceride, mmol/L	1.5 [1.0, 2.3]	1.4 [1.0, 1.9]	1.5 [1.0, 2.3]	1.4 [1.0, 2.2]	1.7 [1.1, 2.5]	0.057	1.5 [1.0, 2.5]	1.5 [1.0, 2.2]	1.4 [1.0, 2.0]	1.5 [1.0, 2.3]	0.410
Cholesterol, mmol/L	4.1 ± 1.0	4.0 ± 0.90	4.1 ± 1.0	4.2 ± 1.0	4.0 ± 1.0	0.128	4.2 ± 0.9	4.0 ± 0.9	4.1 ± 1.0	4.1 ± 1.0	0.046
HDL-c, mmol/L	1.2 ± 0.4	1.3 ± 0.5	1.2 ± 0.42	1.2 ± 0.4	1.2 ± 0.4	0.008	1.3 ± 0.5	1.2 ± 0.4	1.2 ± 0.4	1.2 ± 0.4	0.328
LDL-c, mmol/L	2.4 ± 0.9	2.4 ± 1.0	2.5 ± 0.9	2.4 ± 1.0	2.4 ± 0.9	0.652	2.6 ± 1.0	2.4 ± 0.9	2.4 ± 1.0	2.3 ± 0.9	0.021
Anthropometric											
BMI (kg/m^2^)	23.1 ± 3.8	22.4 ± 3.5	23.2 ± 4.0	23.1 ± 3.7	23.6 ± 3.5	0.006	23.6 ± 3.8	22.9 ± 3.5	22.9 ± 3.6	23.0 ± 3.9	0.119
1-year change of BMI (%)	0.0 (− 3.2, 4.3)	− 0.6 (− 4.4, 3.4)	− 0.6 (− 4.5, 2.7)	0.4 (− 2.5, 5.2)	1.6 (− 1.7, 7.0)	< 0.001	− 2.7 (− 7.7, 0.4)	3.5 (− 2.4, 3.5)	2.5 (− 1.1, 7.9)	0.4 (− 2.7, 4.8)	< 0.001
WHtR	0.92 (0.85, 0.99)	0.91 (0.84, 0.98)	0.92 (0.86, 0.98)	0.92 (0.86, 0.98)	0.93 (0.86, 0.99)	0.418	0.93 (0.87, 1.00)	0.92 (0.85, 0.99)	0.92 (0.86, 0.98)	0.92 (0.86, 0.98)	0.214
1-year change of WHtR (%)	0.5 (− 5.6, 7.3)	− 0.1 (− 6.8, 7.6)	1.0 (− 5.2, 6.8)	0.5 (− 5.0, 6.3)	0.6 (− 4.3, 8.8)	0.531	− 0.9 (− 8.4, 7.0)	1.0 (− 5.8, 7.7)	1.6 (− 3.7, 6.7)	0.5 (− 4.6, 7.3)	0.054
TSF, mm	10.0 ± 4.2	11.6 ± 4.7	10.4 ± 4.3	10.0 ± 3.8	7.9 ± 2.9	< 0.001	10.5 ± 4.5	9.9 ± 3.8	9.6 ± 4.2	9.8 ± 4.1	0.124
1-year change of TSF (%)	0.1 (− 25.5, 30.4)	− 41.2 (− 50.0, − 30.4)	− 12.5 (− 20.0, − 6.7)	12.5 (6.1, 20.9)	69.2 (42.6, 106.3)	< 0.001	− 15.8 (− 40.0, 10.0)	− 3.8 (− 25.0, 25.0)	19.0 (− 10.0, 59.3)	2.3 (− 22.2, 28.6)	< 0.001
MUAC, cm	24.6 ± 2.9	24.1 ± 2.8	24.8 ± 3.0	24.6 ± 2.9	24.8 ± 2.9	0.017	25.9 ± 3.2	24.7 ± 2.7	23.6 ± 2.4	24.2 ± 2.8	< 0.001
1-year change of MUAC (%)	− 0.4 (− 6.1, 5.0)	− 3.9 (− 10.0, 3.4)	− 3.1 (− 6.8, 2.4)	1.3 (− 2.3, 7.5)	0.8 (− 2.9, 4.4)	< 0.001	− 10.6 (− 14.6, − 7.7)	− 3.2 (− 4.4, − 1.8)	1.9 (0.0, 3.4)	10.2 (7.1, 14.2)	< 0.001
MAMC, cm	21.4 ± 2.7	20.5 ± 2.5	21.5 ± 2.5	21.5 ± 2.6	22.4 ± 2.7	< 0.001	22.6 ± 3.0	21.6 ± 2.6	20.5 ± 2.1	21.1 ± 2.5	< 0.001
MIS	6.5 ± 2.9	6.2 ± 2.2	6.5 ± 2.8	6.5 ± 2.8	6.7 ± 3.6	0.813	6.4 ± 2.8	6.7 ± 2.6	6.4 ± 3.3	6.2 ± 2.9	0.703
Outcomes											
Follow-up duration, months	48.0 (37.0, 59.0)	48.0 (36.0, 59.0)	48.0 (37.0, 59.0)	49.0 (36.5, 59.0)	49.0 (45.5, 60.0)	0.085	48.0 (36.0, 59.0)	57.0 (42.0, 61.0)	48.0 (36.0, 59.0)	49.0 (39.0, 59.0)	0.137
All-cause mortality n (%)	206 (21.2%)	74 (30.5%)	51 (21.0%)	46 (18.8%)	35 (14.5%)	< 0.001	70 (28.8%)	52 (21.4%)	49 (19.8%)	35 (14.6%)	0.002

Data are presented as mean (standard deviation) or median (25th, 75th centile) for continuous variables, and percentages for categorical variables. Categorical variables were compared using the chi-square test. Continuous variables were compared using ANOVA and Kruskal–Wallis tests. *P*-value < 0.05 presents statistical difference.

*TSF* triceps skinfold, *MUAC* mid-upper arm circumference, *HD* hemodialysis, *CVD* cardiovascular disease, *BMI* body mass index, *WHtR* Waist-hip ratio, *MAMC* mid-arm muscle circumference, *CAMA* correlated arm muscle area, *SBP* systolic blood pressure, *DBP* diastolic blood pressure, *mCCI* modified Charlson comorbidity index, *PTH* parathyroid hormone, *HDL-c* high density lipoprotein-cholesterol, *LDL-c* low density lipoprotein-cholesterol, *MIS* malnutrition-inflammation score.

During the follow-up for a median of 48.0 (37.0, 59.0) months, 206 (21.2%) all-cause mortality events were recorded. Compared with the lowest quartile of TSF trajectory, HD patients with a higher quartile had a lower risk of all-cause mortality (*P* < 0.05). Patients stratified by MUAC trajectory had similar results (Table 1).

### Association between all-cause mortality and TSF, MUAC trajectories

**Figure 2:**
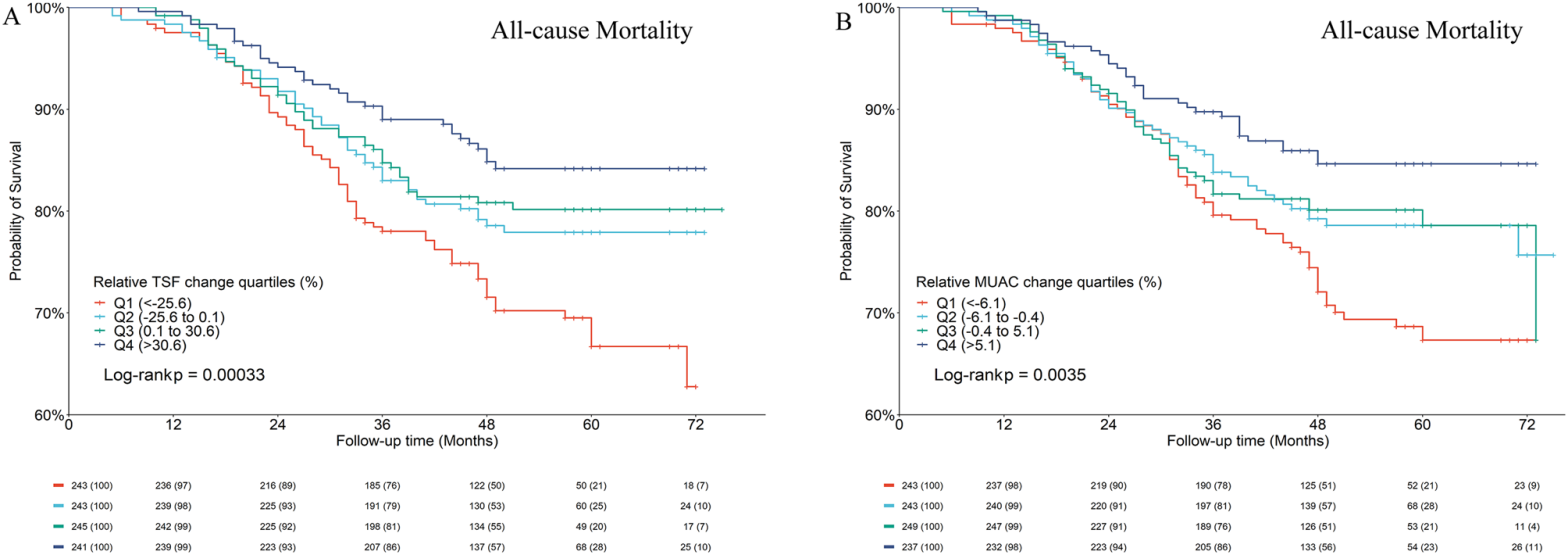
Kaplan–Meier analyses for the incidence of all-cause mortality in patients receiving hemodialysis according to the TSF and MUAC trajectory. Patients were divided into 4 groups according to percent change quartiles of TSF and MUAC. TSF, triceps skinfold; MUAC, mid-upper arm circumference.

For both TSF and MUAC trajectories, Kaplan–Meier analyses revealed a lower risk of all-cause mortality in the highest quartile group (Fig. 2), and the log-rank statistics showed significant differences in survival time among groups (*P* < 0.001).

Table 2 shows the association between TSF, and MUAC trajectories and subsequent all-cause mortality. In the multivariable-adjusted Cox regression analysis, HD patients in the Q2 group of TSF trajectory (HR = 0.569 [95% CI 0.386–0.838], *P* = 0.004), Q3 group (HR = 0.568 [95% CI 0.387–0.836], *P* = 0.004), and Q4 group (HR = 0.405 [95% CI 0.257–0.640], *P* < 0.001) all had a lower risk of all-cause mortality, compared with those in Q1 group (Table 2). Compared with the Q1 group of MUAC trajectory, only HD patients in the Q3 (HR = 0.525, 95% CI 0.345–0.799, *P* = 0.003), and Q4 group (HR = 0.537, 95% CI 0.345–0.837, *P* = 0.006) had a significantly lower risk of all-cause mortality (Table 2). Even adjusting for baseline BMI, TSF, MUAC, and BMI trajectory, these significant associations could be still observed. Furthermore, comparable results were found when MUAC and TSF trajectory was included as a confounder in the mutual models for TSF and MUAC trajectory, respectively.

**Table 2:** Cox regression analysis of all-cause mortality with TSF and MUAC trajectories in HD patients.

Variables	Unadjusted		Model 1^a^		Model 2 ^b^		Model 3 ^c^		Mutual model ^d^	
	HR (95%CI)	*P*	HR (95%CI)	*P*	HR (95%CI)	*P*	HR (95%CI)	*P*	HR (95%CI)	*P*
TSF trajectory										
Continuous (1-SD)	0.666 (0.554, 0.802)	< 0.001	0.642 (0.532, 0.774)	< 0.001	0.472 (0.336, 0.663)	< 0.001	0.443 (0.302, 0.649)	< 0.001	0.555 (0.380, 0.810)	0.002
Quartiles										
Q1 (≤ − 25.6%)	**Reference**		**Reference**		**Reference**		**Reference**		**Reference**	
Q2 (− 25.6% to 0.1%)	0.663 (0.464, 0.947)	0.024	0.614 (0.430, 0.878)	0.007	0.569 (0.389, 0.834)	0.004	0.569 (0.386, 0.838)	0.004	0.650 (0.438, 0.965)	0.032
Q3 (0.1% to 30.6%)	0.601 (0.416, 0.868)	0.007	0.593 (0.410, 0.856)	0.005	0.573 (0.396, 0.828)	0.003	0.568 (0.387, 0.836)	0.004	0.686 (0.461, 1.021)	0.063
Q4 (≥ 30.6%)	0.444 (0.297–0.665)	< 0.001	0.409 (0.274,0.612)	< 0.001	0.415 (0.276,0.624)	< 0.001	0.405 (0.257, 0.640)	< 0.001	0.524 (0.326, 0.842)	0.008
*P* for trend	< 0.001		< 0.001		< 0.001		< 0.001		0.001	
Cutoff value										
C2*	0.640 (0.485, 0.845)	0.002	0.634 (0.480, 0.837)	0.001	0.638 (0.480, 0.847)	0.002	0.661 (0.485, 0.899)	0.008	0.776 (0.566, 1.064)	0.116
MUAC trajectory										
Continuous (1-SD)	0.072 (0.020, 0.258)	< 0.001	0.095 (0.025, 0.360)	< 0.001	0.084 (0.021, 0341)	0.001	0.022 (0.005, 0.102)	< 0.001	0.038 (0.007, 0.215)	< 0.001
Quartiles										
Q1 (≤ − 6.1%)	**Reference**		**Reference**		**Reference**		**Reference**		**Reference**	
Q2 (− 6.1% to -0.4%)	0.714 (0.499, 1.023)	0.066	0.812 (0.566, 1.163)	0.256	0.814 (0.561, 1.180)	0.277	0.716 (0.490, 1.047)	0.085	0.801 (0.543, 1.181)	0.263
Q3 (− 0.4% to 5.1%)	0.682 (0.473, 0.9883)	0.040	0.750 (0.520, 1.082)	0.124	0.619 (0.413, 0.927)	0.020	0.525 (0.345, 0.799)	0.003	0.615 (0.397, 0.952)	0.029
Q4 (≥ 5.1%)	0.493 (0.328, 0.739)	0.001	0.531 (0.354, 0.797)	0.002	0.682 (0.455, 1.024)	0.064	0.537 (0.345, 0.837)	0.006	0.710 (0.442, 1.139)	0.155
*P* for trend	0.006		0.023		0.020		0.035		0.088	
Cutoff value										
C2*	0.693 (0.525, 0.916)	0.010	0.732 (0.552, 0.971)	0.031	0.732 (0.547, 0.980)	0.036	0.654 (0.479, 0.892)	0.007	0.765 (0.553, 1.058)	0.105

^a^Model 1, adjusted for age, sex (male or female), educational level (low or high), living alone (yes or not), smoking status (ever/current or never smoker), alcohol consumption (yes or no).

^b^Model 2, adjusted for Model 1, systolic blood pressure, diastolic blood pressure, and diabetes, hypertension, cardiovascular disease, dialysis frequency (less than thrice one week or thrice one week and more), hemoglobin level, albumin level, creatinine level, and malnutrition inflammatory score, modified Charlson comorbidity index, and BMI trajectory.

^c^Model 3, adjusted for Model 2, baseline body mass index, TSF, and MUAC.

^d^Mutual model, adjusted for Model 2, and MUAC trajectory or TSF trajectory.

*The cutoff value was calculated as the median of TSF or MUAC trajectory, and C1 were regard as the reference.

*TSF* triceps skinfold, *MUAC* mid-upper arm circumference, *HR* hazard ratio, *CI* confidence interval.

Mid-arm measurements were treated as continuous variables, TSF, and MUAC were consistently and inversely associated with all-cause mortality (Table 2). Representing HRs per 1-SD increase (standardized HR), after full adjustments, a 1-SD increase in relative TSF, and MUAC trajectories were associated with a 44.5%, and 96.2% decreased risk of all-cause mortality, respectively (mutual model).

The median stratification method showed that the cutoff values of the TSF trajectory was 0%, and the MUAC trajectory was − 4.0%. Compared with the C1 group, HD patients in the C2 group of TSF, and MUAC trajectory both had a lower risk of all-cause mortality (HR = 0.661, 0.654, both *P* < 0.005).

### Cross-classified analyses

The associations in the cross-classified analyses are shown in Table 3. Compared with T1 group (both lower TSF and MUAC trajectory), T2, T3 (lower TSF or MUAC trajectory), and T4 (both higher TSF and MUAC trajectory) all had lower risk of all-cause mortality (*P* < 0.05) in the multivariate model. HD patients with higher TSF and MUAC trajectories had a 46.9% decreased risk of all-cause mortality (Table 3).

**Table 3:** Cox regression analysis of mortality according to the cutoff values of TSF and MUAC trajectories in HD patients.

Variables	Unadjusted		Model 1 ^a^		Model 2 ^b^		Model 3 ^c^	
	HR (95% CI)	*P*	HR (95% CI)	*P*	HR (95% CI)	*P*	HR (95% CI)	*P*
*All-cause Mortality*								
T1	**Reference**		**Reference**		**Reference**		**Reference**	
T2	0.598 (0.406, 0.880)	0.009	0.541 (0.366, 0.799)	0.002	0.526 (0.354, 0.781)	0.001	0.570 (0.377, 0.862)	0.008
T3	0.658 (0.442, 0.980)	0.039	0.640 (0.428, 0.958)	0.030	0.613 (0.406, 0.926)	0.020	0.551 (0.358, 0.848)	0.007
T4	0.530 (0.373, 0.752)	< 0.001	0.547 (0.384, 0.780)	0.001	0.545 (0.378, 0.786)	0.001	0.531 (0.361, 0.782)	0.001

^a^Model 1, adjusted for age, sex (male or female), educational level (low or high), living alone (yes or not), smoking status (ever/current or never smoker), alcohol consumption (yes or no).

^b^Model 2, adjusted for Model 1, systolic blood pressure, diastolic blood pressure, and diabetes, hypertension, cardiovascular disease, dialysis frequency (less than thrice one week or thrice one week and more), hemoglobin level, albumin level, creatinine level, and malnutrition inflammatory score, modified Charlson comorbidity index, and BMI trajectory.

^c^Model 3, adjusted for Model 2, baseline body mass index, TSF, and MUAC.

*TSF* triceps skinfold, *MUAC* mid-upper arm circumference, *HR* hazard ratio, *CI* confidence interval.

### RCS analyses

**Figure 3:**
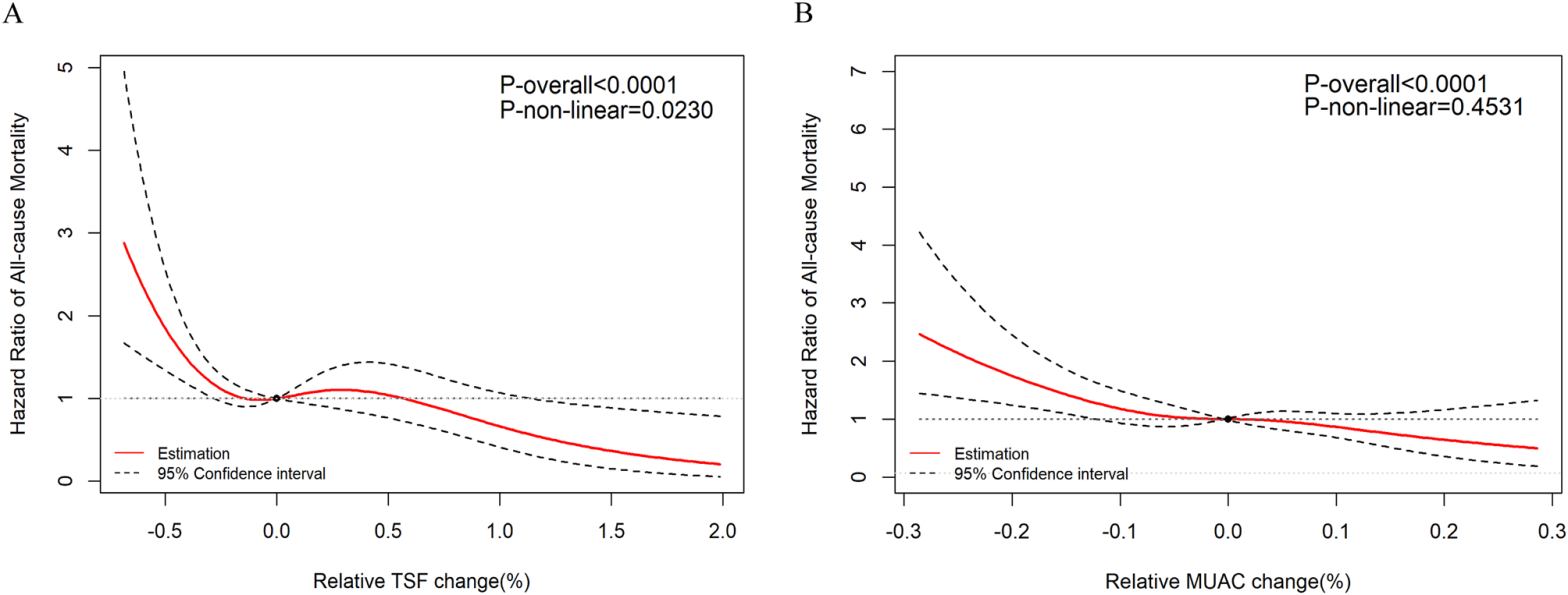
The dose–response relationship between TSF, MUAC trajectory and all-cause mortality in hemodialysis patients. Point estimates (solid line) and 95% confidence intervals (dashed lines) were estimated by restricted cubic splines analysis with knots placed at the 10th, 50th, and 90th percentile. Model was adjusted for age, sex, educational level, living status, smoking status, alcohol consumption, systolic blood pressure, diastolic blood pressure, diabetes, hypertension, cardiovascular disease, dialysis frequency, body mass index trajectory, and MUAC or TSF trajectory. TSF, triceps skinfold; MUAC, mid-upper arm circumference.

In multivariable-adjusted cubic spline analyses showed that all-cause mortality decreased consistently with increasing MUAC trajectory (Fig. 3B), and the spline variable confirmed no departure from the linear relationship (nonlinear *P* = 0.4531) of MUAC trajectory and all-cause mortality. However, the TSF trajectory showed an inverted L-shaped association with all-cause mortality (nonlinear *P* = 0.0230; Fig. 3A).

### Subgroup analyses

**Figure 4:**
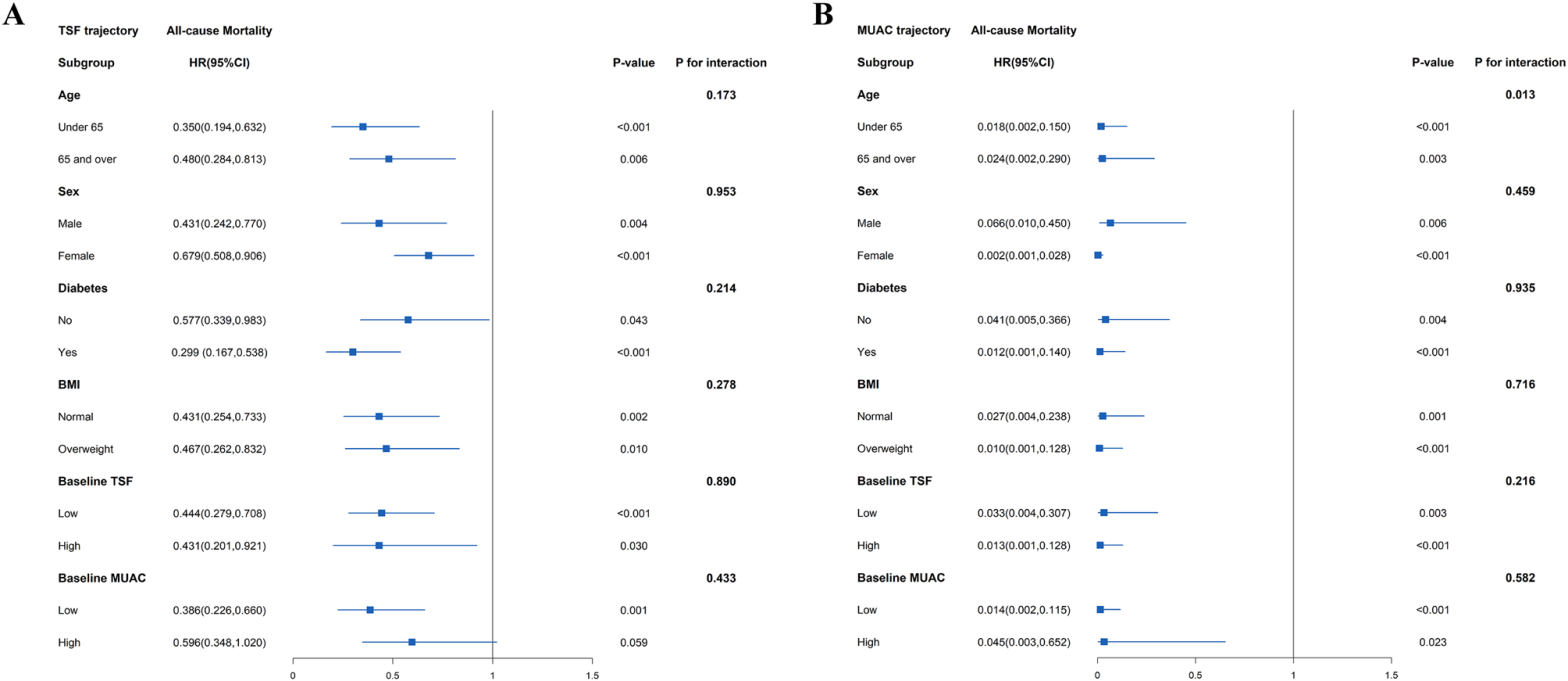
Subgroup analyses of association between TSF, MUAC trajectory and all-cause mortality in hemodialysis patients. Model was adjusted for age, sex, educational level, living status, smoking status, alcohol consumption, systolic blood pressure, diastolic blood pressure, diabetes, hypertension, cardiovascular disease, dialysis frequency, body mass index trajectory, and MUAC or TSF trajectory. TSF, triceps skinfold; MUAC, mid-upper arm circumference; BMI, body mass index; WC, waist circumference; HR, hazard ratio; CI: confidence interval.

This study also performed subgroup analyses to explore potential heterogeneity between TSF, MUAC trajectory, and mortality, stratified by age (< 65 or ≥ 65 years), sex (female or male), diabetes (no or yes), BMI (normal or overweight), WC (normal or central obesity), and TSF or MUAC at baseline (low or high as the median), respectively (Table S1), which are shown in the forest plots (Fig. 4). In any subgroups, TSF and MUAC trajectory were considered as continuous variables with 1-SD. There were significant interactions between MUAC trajectory and age (*P* = 0.013), baseline MUAC (*P* = 0.039), and all-cause mortality (Table S1; Fig. 4). Robust results were found in associations between all-cause mortality and TSF and MUAC trajectory in subgroups, including age < 65, diabetes, central obesity at baseline, and low TSF or MUAC at baseline (Fig. 4).

### Sensitivity analyses

Multivariate-adjusted Fine-Gray sub-distributional hazard models, as sensitivity analyses, revealed similar results (Table S2). Higher changes in TSF (sHR = 0.680, 95% CI: 0.565–0.819, *P* < 0.001), and MUAC (sHR = 0.776, 95% CI: 0.668–0.901, *P* < 0.001) had a significantly lower risk of all-cause mortality, remaining even in the mutual-adjusted model. Similarly, higher changes in TSF (sHR = 0.535, 95% CI: 0.391–0.733, *P* < 0.001).

## Discussion

In this multicenter, prospective cohort study, we investigated the association between the trajectory of mid-arm measurement and mortality in a cohort of patients receiving HD. Results demonstrated that reduction in TSF, and MUAC over a 1-year period is strongly associated with the higher risk of all-cause mortality among HD patients, independent of demographic characteristics, and comorbid conditions. Of note, the reduction in TSF and MUAC were still negatively associated with survival, even after adjusting baseline BMI, TSF, MUAC, and BMI trajectories over a 1-year period. Therefore, these findings underscore the importance of routine monitoring of TSF and MUAC, in order to provide significant prognostic information and will guide interventions to optimize the survival outcomes in HD patients.

A number of studies have proven that free fat is beneficial to the health of humans ^25^, and its depletion is common in HD patients ^26^. The skin is one of the largest organs that store adipose tissue, TSF-reflected mid-arm subcutaneous fat could represent the distribution of peripheral fat well, and its loss is an effective predictor for both inflammation and malnutrition ^27^. TSF thickness has been considered a promising tool for predicting cardiovascular events and mortality risk ^14,15^. For HD patients, Huang et al. also found a significant association between lower quartiles of TSF thickness and higher all-cause mortality in 1709 patients ^16^. Inadequate subcutaneous fat can disturb normal glucose and lipid metabolism and immune response by inhibiting the production of leptin ^28^. The deficiency of subcutaneous tissue adipose would further cause ectopic fatty deposition, inducing chronic inflammation, and insulin resistance ^29^. In addition, insufficient subcutaneous fat can also cause atherosclerosis and non-adipose tissue lipotoxicity by inhibiting the separation of non-esterified fatty acids from food ^30^, potentially increasing all-cause death risk ^31,32^.

This current study found that TSF decrease over a 1-year period was associated with a higher risk of all-cause mortality for the first time. Several studies showed similar results ^21,33^. The study of Kamyar et al. found that fat loss over time, defined as TSF-estimated body fat fraction, was independently associated with higher mortality in HD patients. This study showed that a fat loss (< 1%) was associated with a death risk 2 times that of patients who gained fat (> 1%) (HR:2.06; 95% CI 1.05–4.05; *P* = 0.04) ^33^. However, Hollander et al. showed decreases in TSF had no association with all-cause mortality among the European elderly population of 70- to 77-year-old individuals after adjusting for characteristics ^20^. The different results may depend on that this study analyzed the TSF divided into quintiles and used the smallest change as the reference category, they did not examine the TSF as a continuous variable.

MUAC is a reliable substitution of body mass or muscle mass and is readily a clinically useful indicator of nutritional status ^13^. A decrease in MUAC may primarily reflect loss of muscle mass, leading to protein-energy wasting, sarcopenia, and malnutrition inflammation syndrome. These processes have been proven to be the determinants of CVD and mortality risk ^19,20^. The present result of a decrease in MUAC being associated with increased mortality risk is in line with the previous finding by Schaap et al., in which decreases in MUAC have the strongest association with all-cause mortality ^19^. Moreover, this study focused on the specific population with HD as kidney replacement therapy, which extends the existing conclusions. The current study observed that 1-SD increase in relative MUAC change was inversely associated with risk reductions for all-cause mortality in a consistent linear trend without significant departure, which is not consistent with the findings by De Hollander et al. ^20^. In the study, a decrease, as well as an increase in MUAC, were significantly associated with increased all-cause mortality risk. Our current study did not observe a similar pattern. However, to date, no other studies have reported an association between increasing MUAC and higher mortality risk among HD patients.

Of note, in the current study, after adjusting for BMI trajectory in the Cox hazard analyses, the relationships between TSF, MUAC trajectories, and all-cause mortality were still observed. Although previous studies have proven that a declining body weight, commonly defined with BMI or WC, is associated with higher mortality ^34,35^, some studies demonstrated that BMI and its change were not associated with mortality ^36,37^. Taken the accuracy of BMI trajectory could be influenced by a change in body water balance, BMI might diminish the reproducibility of this measure, weakening the association with mortality. Its interpretation in the assessment of nutritional status could be compromised by the inability to assess the distribution of body composition, which implies that BMI is not a reliable marker of nutritional status in HD patients ^38^. What’s more, the ratio of fat and muscle mass loss in BMI decrease may also vary depending on age and sex ^36^. Recent studies have suggested that TSF and MUAC can be more feasible and more valid measures of free fat and thinness than BMI, respectively ^37,39^.

The current study implies that subcutaneous fat mass and muscle mass trajectories provide additional prognostic information to the BMI trajectory. Additionally, the lack of interaction between baseline and 1-year change in fat mass in terms of mortality suggests that the relative benefit of increasing fat mass, or the deleterious effect of decreasing fat mass, remains consistent irrespective of starting level. Thus, this study provides further justification for evaluating the fat mass trajectory in addition to the BMI trajectory in patients receiving HD. However, we found an interaction between baseline and 1-year change in muscle body in terms of mortality. When initial MUAC is low, a reduction in MUAC is more prominently associated with all-cause mortality. A possible explanation for the result is that decreasing MUAC reflects muscle mass loss and protein energy wasting, and is related to an increased risk of mortality ^40^. Anyway, we found that there were higher prognostic values for the TSF and MUAC changes than for BMI change, which further emphasizes the importance of performing subcutaneous fat and muscle mass assessment for managing the HD population.

We also calculated thresholds of TSF and MUAC trajectories over a 1-year period to facilitate the identification of survival-related low fat or low muscle mass for HD patients. Although a number of studies have emphasized the integrality of the assessments of fat and muscle mass, which can be reflected by the TSF and MUAC respectively, any current nutritional risk screening tools or diagnosing criteria for HD patients have included the two assessments as the main components. Furthermore, the importance of these regular measurements is still seriously underestimated among the HD population. Therefore, we provided the thresholds of TSF and MUAC trajectories for the first time, which may offer reference values for future studies addressing the assessment of the body composition change in clinical settings.

This is the first study to explore the prognostic potential of mid-arm measurement trajectory in mortality for HD patients. These findings are valuable for improving regular nutritional status evaluations and risk stratification. In addition, the long follow-up time of a median of 48 months and the fact that data were obtained from 22 HD units in Southwestern China make it possible to generalize the results to the general HD population in this area. Due to their simplicity and cost performance, regular and reduplicative measurements of TSF and MUAC over time may have critical values as surrogate indicators of changes in fat mass and body mass among HD patients. These findings emphasize the importance of subcutaneous fat and muscle mass assessment to guide strategies to optimize long-term outcomes in HD patients. Our study also suggests that the measurements of TSF and MUAC trajectories may be better methods for assessing nutritional status to predict poor prognosis independent of body weight trajectory. Therefore, in addition to weight loss, clinicians should also consider interventions to improve the fat and muscle mass in HD patients, such as more individualized nutritional supplementation. These may be considered strengths of the current study.

There are several limitations to declare. First, the results of our study were based on the data from the HD population in Southwestern China, and the findings need to be confirmed in other populations. Second, despite a great quantity of potentially confounding factors having been adjusted, and the nature of all observational studies, some undetected and unmeasured confounders still cannot be excluded, such C-reactive protein levels, and oral medications. Third, although muscle mass and soft tissue volume are critical prognostic factors, it was essential to consider muscle mobility and motility as risk factors. However, the quantity and quality of exercise were not evaluated, due to the inconvenience among HD outpatients. Further analyses of muscle parameters are still needed to comprehensively elaborate the effect of muscle on prognosis among HD patients. Finally, TSF and MUAC measurements are commonly considered to have a low reproducibility. To overcome this barrier, this study dispatched the same group of trained research doctors to perform clinical assessments through face-to-face questionnaire interviews and physical measurements. Moreover, baseline and follow-up measurements were performed following standardized protocols for anthropometric measurements, as recommended by the World Health Organization. Therefore, this ensures the reproducibility of TSF and MUAC measurements in a certain extent. Currently, some precise measurements of changes in body composition have been applied in clinical practice, such as dual energy x-ray absorptiometry, CT, MRI, or bioelectrical impedance analysis ^41^, which are more sophisticated than mid-arm measurements. However, the use of these measures is not feasible to popularize for clinical application, due to the complicated operation, expensive, and dependent on facility instruments. Besides, TSF and MUAC can be conveniently measured at smaller institutions and in community settings. Therefore, measurements of body composition represent a practical approach that can balance clinical needs and cost-effectiveness in a wide range of scenarios. Nevertheless, future studies on body composition trajectory are needed to confirm our findings.

In conclusion, we found that decreasing TSF thickness and MUAC over time, are independently associated with higher risk of all-cause mortality, independent of BMI trajectory. The TSF thickness and MUAC trajectories are recommended to be convenient and credible indicators to predict mortality in clinical practice. Further high-quality randomized controlled trials of early intervention of TSF thickness and MUAC are required in patients receiving HD.

### Supplementary Information

Supplementary Information.

## Data Availability

The datasets generated during the current study are available from the corresponding author upon reasonable request.
