# Supplementary material for: Trajectory of mid-arm subcutaneous fat, muscle mass predicts mortality in hemodialysis patients independent of body mass index
Source: Sci Rep. 2024 Jun 18;14:14005. doi: 10.1038/s41598-024-64728-8 (PMC11189518; doi:10.1038/s41598-024-64728-8)
Supplement: Supplementary file 1 — Supplementary Information. [file 41598_2024_64728_MOESM1_ESM.docx]

**Supplementary Material**


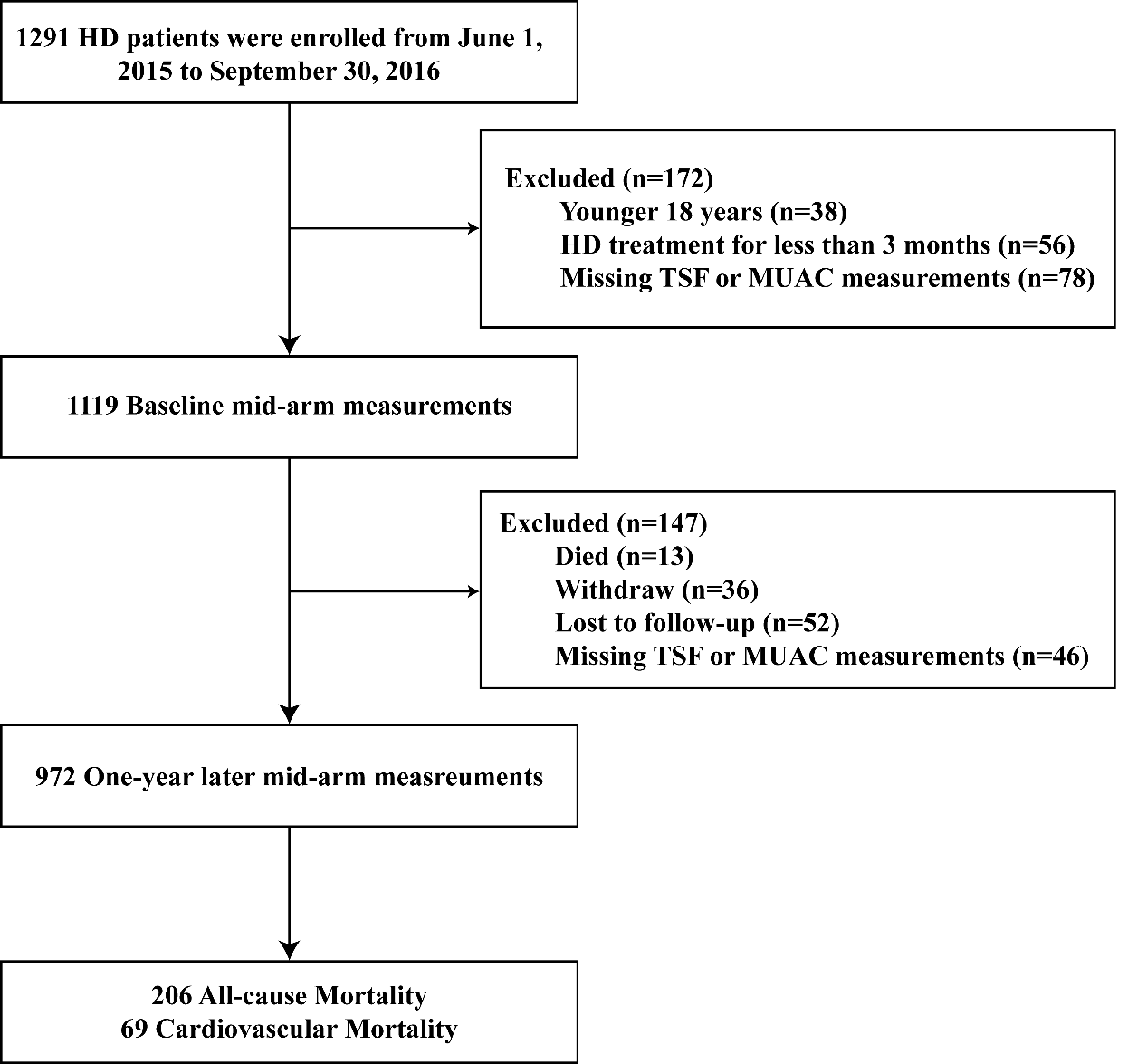


**Figure S1:** Patient selection flowchart. HD, hemodialysis; TSF, triceps skinfold; MUAC, mid-upper arm circumference.

**Table S1. Subgroup analyses of all-cause mortality and 1-SD increase in TSF, and MUAC trajectories in HD patients.**

| **Variables** | **Multivariate analysis^*^** | | | | | |
| --- | --- | --- | --- | --- | --- | --- |
|  | **TSF trajectory** | | | **MUAC trajectory** | | |
|  | **HR(95%CI)** | ***P*-value** | ***P* for interaction** | **HR(95%CI)** | ***P*-value** | ***P* for interaction** |
| **Age categories** |  |  | 0.173 |  |  | 0.013 |
| Age<65 | 0.350(0.194,0.632) | <0.001 |  | 0.018(0.002,0.150) | <0.001 |  |
| Age≥65 | 0.480(0.284,0.813) | 0.006 |  | 0.024(0.002,0.290) | 0.003 |  |
| **Sex** |  |  | 0.953 |  |  | 0.459 |
| Male | 0.431(0.242,0.770) | 0.004 |  | 0.066(0.010,0.450) | 0.006 |  |
| Female | 0.679(0.508,0.906) | <0.001 |  | 0.002(0.001,0.028) | <0.001 |  |
| **Diabetes** |  |  | 0.214 |  |  | 0.935 |
| No | 0.577(0.339,0.983) | 0.043 |  | 0.041(0.005,0.366) | 0.004 |  |
| Yes | 0.299 (0.167,0.538) | <0.001 |  | 0.012(0.001,0.140) | <0.001 |  |
| **BMI** |  |  | 0.278 |  |  | 0.716 |
| Normal | 0.431(0.254,0.733) | 0.002 |  | 0.027(0.004,0.238) | 0.001 |  |
| Overweight | 0.467(0.262,0.832) | 0.010 |  | 0.010(0.001,0.128) | <0.001 |  |
| **Baseline TSF** |  |  | 0.890 |  |  | 0.216 |
| Low | 0.444(0.279,0.708) | <0.001 |  | 0.033(0.004,0.307) | 0.003 |  |
| High | 0.431(0.201,0.921) | 0.030 |  | 0.013(0.001,0.128) | <0.001 |  |
| **Baseline MUAC** |  |  | 0.433 |  |  | 0.582 |
| Low | 0.386(0.226,0.660) | 0.001 |  | 0.014(0.002,0.115) | <0.001 |  |
| High | 0.596(0.348,1.020) | 0.059 |  | 0.045(0.003,0.652) | 0.023 |  |

^*^ adjusted for age, sex (male or female), educational level (low or high), living alone (yes or not), smoking status (ever/current or never smoker), alcohol consumption (yes or no), systolic blood pressure, diastolic blood pressure, and diabetes, hypertension, cardiovascular disease, dialysis frequency (less than thrice one week or thrice one week and more), hemoglobin level, albumin level, creatinine level, and malnutrition inflammatory score, modified Charlson comorbidity index, BMI trajectory, baseline body mass index, TSF, and MUAC.

TSF, triceps skinfold; MUAC, mid-upper arm circumference; HD, hemodialysis; HR, hazard ratio; CI: confidence interval.

**Table S2. Competing risk analysis of all-cause mortality with 1-SD TSF and MUAC trajectories in HD patients.**

| **Variables** | **Unadjusted model** | | **Model 1^a^** | | **Model 2^b^** | | **Mutual model^c^** | |
| --- | --- | --- | --- | --- | --- | --- | --- | --- |
|  | **sHR(95%CI)** | ***P*** | **sHR(95%CI)** | ***P*** | **sHR(95%CI)** | ***P*** | **sHR(95%CI)** | ***P*** |
| **All-cause Mortality** |  |  |  |  |  |  |  |  |
| TSF trajectory | 0.680(0.565,0.819) | <0.001 | 0.644(0.530,0.784) | <0.001 | 0.650(0.534,0.790) | <0.001 | 0.680(0.565,0.819) | <0.001 |
| MUAC trajectory | 0.742(0.644,0.854) | <0.001 | 0.760(0.657,0.880) | <0.001 | 0.744(0.640,0.864) | <0.001 | 0.776(0.668,0.901) | <0.001 |

^a^ Model 1, adjusted for age, sex (male or female), educational level (low or high), living alone (yes or not), smoking status (ever/current or never smoker), alcohol consumption (yes or no).

^b^ Model 2, adjusted for Model 1, systolic blood pressure, diastolic blood pressure, and diabetes, hypertension, CVD, dialysis frequency (less than thrice one week or thrice one week and more), baseline body mass index trajectory, and waist-to-hip ratio trajectory.

^c^ Mutual model, adjusted for Model 2, and MUAC trajectory or TSF trajectory.

TSF, triceps skinfold; MUAC, mid-upper arm circumference; sHR, sub-distribution hazard ratio; CI: confidence interval.
